# A national survey of antimicrobial stewardship content in Canadian entry-to-practice pharmacy programs

**DOI:** 10.1017/ash.2023.155

**Published:** 2023-05-03

**Authors:** Jenna M. Sauve, Linda D. Dresser, Marie A. Rocchi, Miranda So

**Affiliations:** 1 Leslie Dan Faculty of Pharmacy, University of Toronto, Toronto, Ontario, Canada; 2 University Health Network, Toronto, Ontario, Canada; 3 Toronto General Hospital Research Institute, Toronto, Ontario, Canada

## Abstract

**Objective:**

To describe the current landscape of antimicrobial stewardship (AMS) instruction in Canadian entry-to-practice pharmacy programs and the perceived barriers and facilitators to optimizing teaching and learning.

**Design:**

Electronic survey.

**Participants:**

Faculty representatives from the 10 Canadian entry-to-practice pharmacy programs, including content experts and faculty leadership.

**Methods:**

A review of international literature pertaining to AMS in pharmacy curricula informed a 24-item survey, which was open for completion from March to May of 2021. Curriculum content questions were developed using AMS topics recommended by pharmacy educators in the United States, and professional roles described by the Association of Faculties of Pharmacy of Canada.

**Results:**

All 10 Canadian faculties returned a completed survey. All programs reported teaching AMS principles in their core curricula. Content coverage varied, with programs teaching, on average, 68% of the recommended AMS topics from the United States. Potential gaps were identified within the professional roles of “communicator” and “collaborator.” Didactic methods of content delivery and student assessment, such as lectures and multiple-choice questions, were most frequently used. Three programs offered additional AMS content in their elective curricula. Experiential rotations in AMS were commonly offered, though teaching AMS in formalized interprofessional settings was rare. Curricular time constraints were identified by all programs as a barrier to enhancing AMS instruction. A course to teach AMS, a curriculum framework, and prioritization by the faculty’s curriculum committee were perceived as facilitators.

**Conclusions:**

Our findings highlight potential gaps and areas of opportunity within Canadian pharmacy AMS instruction.

Antimicrobial resistance (AMR) has been referred to as a “silent pandemic” that poses a major threat to global health.^1^ AMR is projected to account for ∼10 million deaths annually by the year 2050 unless significant action is taken.^2^ In Canada, recent estimates indicate that 1 in 16 patients admitted to hospital will develop a multidrug-resistant infection, and 40% of deaths from bacterial infections can be directly attributed to AMR.^3,4^ The 2017 pan-Canadian Framework for Action identified antimicrobial stewardship (AMS) as a key strategy for tackling AMR.^5^ Successful implementation of AMS interventions relies on a well-trained and empowered workforce.^2^ Correspondingly, the framework identified an enhanced and coordinated educational AMS curricula for prescribers, dispensers, and end users of antimicrobials as an opportunity for action.^5^

The role of pharmacists in AMS interventions in the inpatient setting has been well-established.^6,7^ However, with 89.8% of antimicrobial doses in Canada dispensed in the community setting, pharmacists at all practice sites are required to apply AMS principles to patient care.^8^ Given that the majority of pharmacists in Canada do not pursue formal pharmacy training or education beyond their entry-to-practice degree, they will be reliant on the AMS knowledge and skills acquired in the pharmacy curriculum. It is therefore imperative that a foundational AMS curriculum be offered to ensure that graduates can employ this clinical skill set. Although perspectives of Canadian students on this matter have not been explored, 89% of Doctor of Pharmacy graduates in the United States have expressed a desire for more education on appropriate antimicrobial use.^9^

Despite the recognized value of pharmacists in AMS and the Canadian government’s desire to enhance health professions education in this area, there is currently no requirement for entry-to-practice pharmacy programs to incorporate AMS content into their core curricula in Canada. The nonprescriptive nature of the Association of Faculties of Pharmacy of Canada (AFPC) *Educational Outcomes for First Professional Degree Programs in Pharmacy in Canada 2017* gives programs the flexibility to structure their curricula in a way that achieves overall outcomes.^10^ Each of the 10 faculties decides whether AMS content will be included in their curriculum and the extent and nature of this content. Thus, the degree to which AMS is incorporated into entry-to-practice pharmacy curricula remains unknown, as do the methods used for teaching and assessment. To inform best practices for AMS pharmacy education, we must first determine the current status.

The primary objective of this study was to describe the current landscape of AMS instruction in Canadian entry-to-practice pharmacy programs, as characterized by the depth and breadth of content, in addition to methods of content delivery and student assessment. The secondary objective was to identify perceived barriers and facilitators to optimizing AMS instruction.

## Methods

### Survey creation

**Fig. 1.**
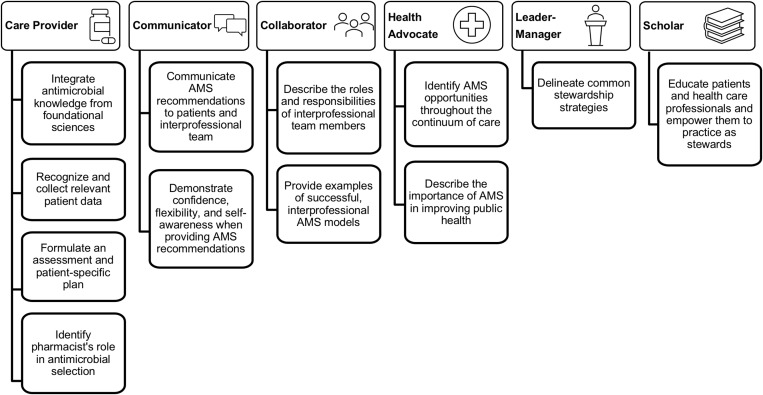
Mapping of antimicrobial stewardship (AMS) topics developed by Gallagher et al^15^ to professional roles as defined by the Association of Faculties of Pharmacy of Canada.^10^

We conducted an electronic survey of AMS content in Canadian entry-to-practice pharmacy curricula. International literature pertaining to pharmacy AMS curricula was reviewed to inform this 24-question survey. Prior surveys by Kufel et al,^11^ Castro-Sachez et al,^12^ and Khan et al^13^ were used to design survey domains and generate survey questions. The 5 survey domains were (1) program characteristics, (2) AMS content and delivery in core curriculum, (3) AMS in elective curriculum, (4) interprofessional and experiential education, and (5) perceived barriers and facilitators to enhancing AMS instruction. For the survey, core curriculum was defined as “program content that all students must satisfy as part of degree requirements.” Questions relating to content delivery and methods for student assessment were adapted from the World Health Organization’s *Curricula Guide for Healthcare Workers Education and Training on Antimicrobial Resistance*.^14^ Questions relating to curriculum content were developed based on topics created by pharmacy educators in the United States in Gallagher et al’s *Preventing the Post-Antibiotic Era by Training Future Pharmacists as Antimicrobial Stewards*.^15^ This reference was used because there are no Canadian consensus standards to serve as a benchmark. To ensure alignment with the educational outcomes of Canadian pharmacy programs, topics were mapped by the project team to professional roles for pharmacists as described in *Educational Outcomes for First Professional Degree Programs in Pharmacy in Canada 2017* (Fig. 1).^10^

The survey instrument was developed using SimpleSurvey (OutSideSoft Solutions, Quebec, Canada, www.simplesurvey.com). Field testing for survey clarity and optimization of design was conducted with a group of interdisciplinary clinicians and clinician educators at University Health Network, as well as 3 pharmacists from external organizations practicing AMS in institutional and ambulatory care settings. Feedback from reviewers was incorporated prior to the final survey iteration. The survey was open for completion from March to May 2021. A waiver of the requirement for Research Ethics Board approval was obtained by University Health Network’s Quality Improvement Review Committee.

### Participants

Prospective survey respondents were identified in consultation with a contact at AFPC. At the time of the survey, all Canadian programs were offering a Doctor of Pharmacy (PharmD) degree, with the exception of 2 programs that were in the process of transitioning from a baccalaureate pharmacy degree to a PharmD program. Two groups of individuals from each university were identified and invited to participate: (1) content experts teaching infectious diseases and/or AMS curriculum content, and (2) individuals in leadership roles, such as associate dean or chair of the curriculum committee, with extensive knowledge of their program’s curriculum structure. Prospective participants were contacted via email to request their participation and were encouraged to work collaboratively within their institution to submit a joint response on behalf of their program.

### Data analysis

All completed surveys were included in the analysis. Questionnaire data were deidentified and findings were aggregated. Categorical data are represented as proportions, and continuous data are represented as measures of central tendency.

## Results

### Overview of AMS in core and elective curricula

A completed survey response was received from each of the 10 pharmacy schools in Canada. All programs identified the presence of AMS content in their core curriculum, and 3 programs reported additional content in the elective curriculum. Core curriculum content was most often covered in the second year of the program, whereas elective content was offered in the third or fourth year. The median number of contact hours devoted to AMS content across the span of the entire core curriculum was 3.25 hours (range, 0.66–65). Among programs offering elective AMS content, the median number of contact hours was 21 (range, 9–26).

### AMS content

The AMS content being taught varied among programs (Table 1). On average, 68% of topics described in Gallagher et al^15^ were taught in the core curriculum; only 1 program taught all topics. Topics mapped to the professional role of care provider were most consistently taught, whereas topics mapped to communicator and collaborator roles were least consistently taught. The topic that was least often covered in the core curriculum was “demonstrate confidence, flexibility, and self-awareness, particularly when providing AMS recommendations.” Programs that offered AMS content in the elective curriculum were covering, on average, 72% of topics described by Gallagher et al (Table 1).^15^

**Table 1.** Antimicrobial Stewardship Topics Taught in the Core and Elective Curricula of Canadian Entry-To-Practice Pharmacy Programs

AMS Learning Objectives^a^ Classified by Professional Role^b^	Core Curriculum^c^ (N = 10)	Elective curriculum^d^ (N = 3)
**Care provider**		
Integrate antimicrobial knowledge from the foundational sciences.	10	3
Recognize and collect relevant patient data.	9	2
Formulate an appropriate assessment and patient-specific plan.	9	2
Identify the role of the pharmacist in antimicrobial selection.	9	3
**Communicator**		
Communicate AMS recommendations to patients and interprofessional team.	6	2
Demonstrate confidence, flexibility, and self-awareness, particularly when providing AMS recommendations.	2	2
**Collaborator**		
Describe the roles and responsibilities of interprofessional team members.	4	2
Provide examples of successful, interprofessional AMS models in various practice settings.	5	1
**Health advocate**		
Identify opportunities for AMS throughout the continuum of care.	7	3
Describe the importance of AMS in improving public health.	9	2
**Leader-Manager**		
Delineate common stewardship strategies.	6	2
**Scholar**		
Educate patients and health care professionals and empower them to practice as stewards.	6	2

Note. AMS, antimicrobial stewardship.

a AMS topics are derived from *Preventing the Post-Antibiotic Era by Training Future Pharmacists as Antimicrobial Stewards*.^15^

b Topics are classified by professional roles as defined in *AFPC Educational Outcomes for First Professional Degree Programs in Pharmacy in Canada 2017*.^10^

c Core curriculum was defined as program content that all students must satisfy as part of degree requirements.

d Elective curriculum was defined as program content that not all students are required to satisfy as part of degree requirements.

### Teaching and assessment methods

Programs used a median of 2 different teaching methods to deliver AMS content in the core curriculum. The most common methods were lectures and large group discussions with 20 or more students (Table 2). Programs with an elective curriculum used 4–5 different methods to deliver AMS content. A median of 2 different assessment methods were used in the core curriculum to evaluate AMS knowledge, skills, and attitudes (Table 3). Multiple-choice or single best-answer questions were used most often, followed by written assessments. Programs with elective curricula used up to 4 different methods for student assessment.

**Table 2.** Teaching Methods Used in Canadian Entry-to-Practice Pharmacy Programs to Deliver Antimicrobial Stewardship Content

Teaching Method	Core Curriculum^a^ (N = 10), No.	Elective Curriculum^b^ (N = 3), No.
Lectures	10	2
Large group facilitated discussions (≥20 students)	5	2
Small group facilitated discussions (<20 students)	1	2
Student presentations or peer-to-peer instruction	0	1
Simulation and role-playing	2	2
E-learning modules (eg, online courses and webinars)	1	0
Project-based learning, by creation of project reports, strategic papers, or critical appraisal of literature	0	1
Other	1	1

a Core curriculum was defined as program content that all students must satisfy as part of degree requirements.

b Elective curriculum was defined as program content that not all students are required to satisfy as part of degree requirements.

**Table 3.** Methods of Assessment Used in Canadian Entry-to-Practice Pharmacy Programs to Assess Students’ Knowledge, Skills and Attitudes Towards Antimicrobial Stewardship

Assessment Method	Core Curriculum (N = 10), No.^a^	Elective Curriculum (N = 3), No.^b^
Multiple-choice/single best-answer questions	10	2
Written assessments (eg, essays, written answer questions, pharmacotherapy care plans)	8	3
Oral assessments (eg, presentations, video assignments, oral examinations)	2	2
Assessment of group discussions	1	1
Other	1	0

a Core curriculum was defined as program content that all students must satisfy as part of degree requirements.

b Elective curriculum was defined as program content that not all students are required to satisfy as part of degree requirements.

### Interprofessional and experiential learning

Only 1 program reported that students could learn AMS content in an interprofessional setting, alongside students from other health professions. Of the 10 programs, 8 programs reported opportunities for AMS-focused experiential rotations. Most commonly, these experiences were offered in the final program year.

### Barriers and facilitators to optimizing AMS instruction

A universally recognized barrier to increasing the depth and breadth of AMS instruction is a lack of time in the curriculum. Other common barriers included the lack of a clear curriculum framework for AMS and other topics in the curriculum having higher priority than AMS (Table 4). Potential facilitators focused on more designated time for AMS and more guidance for content inclusion and delivery (Table 4).

**Table 4.** Perceived Barriers and Facilitators to Increasing the Depth and Breadth of Antimicrobial Stewardship Content as Reported by Content Experts and Faculty Leadership from Canadian Entry-to-Practice Pharmacy Programs

Barriers and Facilitators	Programs Reporting (N = 10), No.
**Barriers**	
Lack of time available in the curriculum	10
Other higher priority topics in the curriculum	3
Lack of a curriculum framework for AMS	3
Lack of instructor(s) with content expertise	0
Belief that AMS is a topic best learned in experiential rotations	0
Belief that AMS is more relevant as a continuing education topic	0
Real or perceived lack of student interest in the topic	0
Other • Lack of a formalized AMS program in the region/province • Lack of integration of AMS principles in infectious diseases curriculum	1 1
**Facilitators**	
Having a course designated to AMS	5
Having a curriculum framework for AMS	4
Having AMS recognized as a priority by faculty’s curriculum committee	4
Guidance on incorporation of AMS into pre-existing curricular structure	3
Other • Repeat exposure to AMS concepts across the curriculum • Individuals who can teach all infectious diseases topics through an AMS lens	1 1

Note. AMS, antimicrobial stewardship.

## Discussion

### Interpretation of findings

Pharmacists are well positioned to engage in AMS interventions in a variety of practice settings. In addition to their traditional dispensing role, expanding the role of pharmacists in prescribing for minor ailments furthers their ability to influence antimicrobial use.^16^ In the current survey results, all programs taught AMS content in their core curriculum, which is encouraging given that inclusion in the core curriculum guarantees exposure for all students. This finding is consistent with surveys conducted in the United States and the United Kingdom, in which most programs included AMS content in the core curriculum.^11,12^ However, our survey results showed that the content was not consistent or comprehensive. On average, programs taught 68% of previously described AMS topics. This variability in content has also been described in the United Kingdom, where pharmacy programs taught 61.5% of the AMS topics included in a questionnaire.^12^ However, a direct comparison is difficult because the UK questionnaire included mostly task-oriented AMS topics.

In our survey results, Canadian programs consistently taught topics within the care provider role, that is, topics that relate to the provision of direct patient care. Because the care provider role is described as the core of the discipline of pharmacy, it is encouraging that pharmacy graduates are taught the necessary content to fulfill this role.^10^ However, to provide quality care, pharmacy graduates are expected to be able to integrate additional professional roles into their practice, such as communicator, collaborator, leader–manager, scholar, and health advocate.^10^ Communication and collaboration skills are critical for pharmacist participation in interdisciplinary AMS discussions and interventions, as well as patient counselling. With many programs not covering topics that fulfill these important roles, a potential gap exists in the readiness of pharmacy graduates to apply their knowledge and patient care skills.

Gaps in the roles of communicator and collaborator likely reflect the teaching of AMS content and assessment of learning. Programs are predominantly using didactic methods of content delivery and student assessment, such as lectures and multiple-choice questions, respectively. Both approaches could limit students’ ability to develop effective communication and collaboration skills. A scoping review of literature surrounding AMS teaching in pharmacy curricula has endorsed active learning as the preferred strategy for the achievement of the necessary learning outcomes.^17^ Incorporation of active learning activities, such as simulation, role-playing, and small group discussions, may foster the development and facilitate the assessment of communication and collaboration skills.

Outside the United Kingdom, which reported a median of 12 hours dedicated to AMS instruction, surveys in other countries have identified that most pharmacy programs dedicate <5 contact hours to AMS in the required curriculum.^11–13^ These findings are similar to those of our survey, in which 8 of the 10 programs reported spending ≤5 hours teaching AMS content in the core curricula. Khan et al^13^ recommended that a minimum of 1–4 hours be devoted to AMS instruction, but they suggested that a greater number of hours (eg, 10–19) would likely be more beneficial, if permitted.

Although the median number of contact hours was low, a vast range of 0.66–65 hours was reported. This expansive range was also reported among pharmacy programs in the United Kingdom, where an interquartile range of 7–25 contact hours was identified.^12^ The heterogeneity in this metric may reflect different approaches to delivering an AMS curriculum. Some programs may devote a limited yet focused number of hours to AMS-specific instruction, whereas others may aim to integrate AMS principles into infectious diseases courses or other components of the curriculum, such as laboratory simulations. Further research is needed to determine the optimal approach for integration of AMS content.

In our study, programs offering an elective course in AMS were able to offer more contact hours for AMS instruction and to employ diverse teaching strategies and assessment methods. Although offering content in elective courses can be a valuable way to enhance student learning, we advocate greater inclusion of AMS content in the core curriculum. Elective content can be offered to students interested in furthering their knowledge in this area; however, having a strong foundational AMS curriculum in required courses will ensure that all graduates can provide patient care with a focus on AMS.

It has been previously demonstrated that an interprofessional AMS curriculum improves knowledge and attitudes toward antimicrobial use among both pharmacy and medical students.^18^ Nevertheless, in our survey, only 1 Canadian program offered the opportunity to learn AMS content alongside students from other health professions. AMS experts have noted that skills in interprofessional interaction are deficient among pharmacy students and practicing pharmacists.^13^ Expanding opportunities for interprofessional education could be a means of addressing gaps in the development of communicator and collaborator roles.

Similar to interprofessional education, experiential education opportunities can enhance AMS-related knowledge and skills, including interprofessional collaboration. It is encouraging that most programs offer student placements with a focus on AMS; however, these opportunities are not available to all students. A lack of preceptor availability in this field has been identified.^11^ With limited availability of AMS-specific rotations, programs and preceptors are encouraged to incorporate AMS teaching into other experiential practice rotations, given that antimicrobials are prescribed throughout the continuum of care. For example, when an AMS checklist and reflection activity was incorporated into student placements in inpatient, ambulatory, and community pharmacy settings, students’ understanding of AMS techniques increased, and students reported being more likely to implement AMS techniques in their careers.^19^

The most commonly perceived barrier to enhancing AMS instruction was a lack of time in the curriculum, which was similarly reported in a recent survey exploring cannabis content in Canadian pharmacy curricula.^20^ An additional barrier raised by one respondent was the “lack of integration of AMS principles in the rest of the infectious diseases curriculum.” This finding suggests a need for a standardized set of core AMS competencies, so that instructors can ensure that key AMS principles are taught. Another barrier raised was the “lack of a formalized AMS program in the region/province.” This deficiency could preclude the opportunity for students to engage in AMS interventions in the experiential curriculum, and it further highlights the importance of having this content in the core curriculum. Particularly in areas lacking formalized programs, Canadian pharmacists will need to be well trained and confident in the area of AMS to act as champions for AMS interventions.

### Strengths and limitations

To our knowledge, this was the first survey to examine AMS pharmacy education in Canada. With responses received from all 10 Canadian programs, our findings provide a comprehensive look at what is being done at the national level. Additionally, participants included both content experts and faculty leadership, providing a more holistic view of the AMS curriculum. However, an important limitation of our survey was that questions addressing curriculum content used AMS topics developed in the United States as the benchmark, given the lack of Canadian-specific standards. Nevertheless, those topics were felt to be the best available benchmark, given the many similarities between the landscape of pharmacy education in Canada and the United States and the strong alignment of the AFPC educational outcomes and the Centre for the Advancement of Pharmacy Education educational outcomes.^21^ A second limitation was that our survey was not designed to capture a detailed picture of AMS in the experiential curriculum, which could offer additional opportunities for knowledge and skill development.

### Implications and future directions

The survey findings highlight important gaps in AMS pharmacy education, which course instructors and curriculum designers should address. Overall, little time is devoted to AMS instruction, and the optimal approach to incorporate AMS content is still unknown. Key competencies may not be addressed with the predominant use of didactic methods for content delivery and student assessment. Specifically, learning activities that promote the development of communicator and collaborator roles should be leveraged to enhance graduates’ ability to engage in collaborative AMS interventions. Similarly, opportunities for learning AMS alongside students from other health professions should be explored.

The heterogeneity of content and time devoted to AMS instruction highlights the need for a common AMS curriculum with standardized educational outcomes. Curriculum planning and evaluation of educational interventions in AMS has also been recommended in prior literature.^17^ Curriculum planning has been done to optimize the instruction of pharmacy informatics in entry-to-practice pharmacy curricula through the development of competencies for pharmacy graduates.^22^ A similar approach could be utilized for AMS instruction. The findings from our survey can serve as a baseline for building an AMS competency framework for pharmacy graduates. Our findings also highlight some of the current gaps and barriers to be addressed.

An opportunity remains for further research regarding the synchronization of AMS teaching with other curriculum topics related to resource stewardship and quality improvement. For example, deprescribing and opioid stewardship are other areas in which pharmacists can play an important role, and where similar principles of resource stewardship may apply. Synchronizing the instruction of these topics could be an effective way to reinforce important skills and principles despite curricular time constraints.

In conclusion, pharmacists can make significant contributions to AMS interventions. Therefore, it is essential that pharmacy students develop the knowledge and skills required to employ AMS principles in a variety of practice settings. The findings from this survey have established a baseline for the status of AMS instruction in Canadian entry-to-practice pharmacy programs and can aid in the advancement of AMS pharmacy education.

## Supplementary material

For supplementary material accompanying this paper visit http://dx.doi.org/10.1017/ash.2023.155. click here to view supplementary material
