# Supplementary material for: A national survey of antimicrobial stewardship content in Canadian entry-to-practice pharmacy programs
Source: Antimicrob Steward Healthc Epidemiol. 2023 May 3;3(1):e86. doi: 10.1017/ash.2023.155 (PMC10173284; doi:10.1017/ash.2023.155)
Supplement: Supplementary file 1 [file S2732494X23001559sup001.pdf]

APPENDIX A: SURVEY INSTRUMENT

INTRODUCTION:

Antimicrobial stewardship refers to coordinated interventions designed to promote, improve, monitor and evaluate judicious antimicrobial usage to preserve their future effectiveness and to promote and protect human and animal health (Government of Canada, 2017). With the growing threat of antimicrobial resistance, it is more important than ever that the next generation of health care professionals are well trained and empowered to practice antimicrobial stewardship.

The objective of this survey is to characterize the current landscape of antimicrobial stewardship instruction in Canadian undergraduate pharmacy curricula. The survey should take approximately 15 minutes to complete. Responses will be analyzed and reported in aggregate. Thank you for your participation.

SECTION 1: Program Demographics

1. Please identify the Faculty or School of Pharmacy that you are representing. *(Note: this is for administrative purposes only and will not be reported*) __________

2. How many students graduate from your undergraduate pharmacy program each year?

- ○ < 50
- ○ 50-100
- ○ 101-200
- ○ > 200

SECTION 2: Antimicrobial Stewardship in the Core Curriculum

Questions 3-9 refer only to your pharmacy program's core curriculum, meaning program content that all students must satisfy as part of degree requirements.

3. Is there a course dedicated primarily to antimicrobial stewardship offered in your core curriculum for the 2020/2021 academic year?

- ○ Yes
- ○ No

4. Are components of antimicrobial stewardship incorporated into other courses in your core curriculum for the 2020/2021 academic year?

- ○ Yes
- ○ No

**If respondent answers "No" to both questions 3 and 4, the survey will automatically skip the remainder of this section and continue to SECTION 3: Antimicrobial Stewardship in the Elective Curriculum.*

5. The following question includes a list of "Antimicrobial Stewardship Learning Objectives for Foundational Learners", as described in *Preventing the Post-Antibiotic Era by Training Future Pharmacists as Antimicrobial Stewards* (Table 1, Gallagher et al., 2018). https://www.ncbi.nlm.nih.gov/pmc/articles/PMC6116871/

Learning objectives have been subdivided according to the most relevant role, as described in the *AFPC Educational Outcomes for First Professional Degree Programs in Pharmacy in Canada 2017*.
https://afpc.info/system/files/public/AFPC-Educational%20Outcomes%202017_final%20Jun2017.pdf

Please select all learning objectives that are incorporated into your program's core curriculum.

CARE PROVIDER

- ☐ Integrate antimicrobial knowledge from the foundational sciences, e.g. mechanism of action or spectrum of activity, to evaluate their potential value in individuals and populations.
- ☐ Recognize and efficiently collect relevant patient data, e.g. infectious signs & symptoms.
- ☐ Formulate an appropriate assessment and plan for a specific patient.
- ☐ Identify the role of the pharmacist in empiric and definitive antimicrobial selection.

COMMUNICATOR

- ☐ Communicate antimicrobial stewardship recommendations to patients and members of the interprofessional team in a variety of settings to promote health and wellness for individuals and communities.
- ☐ Demonstrate confidence, flexibility, and self-awareness, particularly when providing antimicrobial stewardship recommendations.

COLLABORATOR

- ☐ Describe the roles and responsibilities of various members of the interprofessional antimicrobial stewardship team.
- ☐ Provide examples of successful, interprofessional antimicrobial stewardship models in various practice settings.

LEADER-MANAGER

- ☐ Delineate common stewardship strategies, e.g. formulary restriction, prospective review and feedback.

HEALTH ADVOCATE

- ☐ Identify opportunities for antimicrobial stewardship throughout the continuum of patient care, including hospital pharmacy, community pharmacy, transitions of care, and long-term care settings.
- ☐ Describe the importance of antimicrobial stewardship in improving public health.

SCHOLAR

- ☐ Educate patients and health care professionals on foundational antimicrobial stewardship concepts and empower them to practice as stewards.

6. Across the span of the entire core curriculum, how many contact hours are devoted to antimicrobial stewardship? (e.g. a 2 hour lecture would count as 2 contact hours) ____________

7. When is antimicrobial stewardship content covered in the core curriculum? Select all that apply.

- ☐ Year 1
- ☐ Year 2
- ☐ Year 3
- ☐ Year 4
- ☐ Year 5 (where applicable)

8. What pedagogies are used to teach antimicrobial stewardship content in the core curriculum? Select all that apply.

- ☐ Didactic lectures
- ☐ Large group interactive discussions (20 or more students)
- ☐ Small group interactive discussions (less than 20 students)
- ☐ Student presentations or peer-to-peer instruction
- ☐ Simulation and role-playing
- ☐ E-learning modules (e.g. online courses and webinars)
- ☐ Project-based learning, by creation of project reports, strategic papers, or critical appraisal of literature

9. Which assessment methods are used in the core curriculum to evaluate students' knowledge, skills and attitudes related to antimicrobial stewardship? Select all that apply.

- ☐ Multiple choice/single best answer questions
- ☐ Written assessments (e.g. essays, written answer questions, pharmacotherapy care plans)
- ☐ Oral assessments (e.g. presentations, video assignments, oral examinations)
- ☐ Assessment of group discussions
- ☐ Other, please specify: ____________________

SECTION 3: Antimicrobial Stewardship in the Elective Curriculum

Questions 10-16 refer only to your pharmacy program's elective curriculum, meaning program content that students are NOT required to satisfy as part of degree requirements.

10. Is there a course dedicated primarily to antimicrobial stewardship offered in your elective curriculum for the 2020/2021 academic year?

- ○ Yes
- ○ No

11. Are components of antimicrobial stewardship incorporated into other courses in your elective curriculum for the 2020/2021 academic year?

- ○ Yes
- ○ No

**If respondent answers "No" to both questions 10 and 11, the survey will automatically skip the remainder of this section and continue to SECTION 4: Interprofessional and Experiential Education.*

12. The following question includes a list of "Antimicrobial Stewardship Learning Objectives for Foundational Learners", as described in *Preventing the Post-Antibiotic Era by Training Future Pharmacists as Antimicrobial Stewards* (Table 1, Gallagher et al., 2018). https://www.ncbi.nlm.nih.gov/pmc/articles/PMC6116871/

Learning objectives have been subdivided according to the most relevant role, as described in the *AFPC Educational Outcomes for First Professional Degree Programs in Pharmacy in Canada 2017*. https://afpc.info/system/files/public/AFPC-Educational%20Outcomes%202017_final%20Jun2017.pdf

Please select all learning objectives that are incorporated into your program's elective curriculum.

CARE PROVIDER

☐ Integrate antimicrobial knowledge from the foundational sciences, e.g. mechanism of action or spectrum of activity, to evaluate their potential value in individuals and populations.

☐ Recognize and efficiently collect relevant patient data, e.g. infectious signs & symptoms.

☐ Formulate an appropriate assessment and plan for a specific patient.

☐ Identify the role of the pharmacist in empiric and definitive antimicrobial selection.

COMMUNICATOR

☐ Communicate antimicrobial stewardship recommendations to patients and members of the interprofessional team in a variety of settings to promote health and wellness for individuals and communities.

☐ Demonstrate confidence, flexibility, and self-awareness, particularly when providing antimicrobial stewardship recommendations.

COLLABORATOR

☐ Describe the roles and responsibilities of various members of the interprofessional antimicrobial stewardship team.

☐ Provide examples of successful, interprofessional antimicrobial stewardship models in various practice settings.

LEADER-MANAGER

☐ Delineate common stewardship strategies, e.g. formulary restriction, prospective review and feedback.

HEALTH ADVOCATE

☐ Identify opportunities for antimicrobial stewardship throughout the continuum of patient care, including hospital pharmacy, community pharmacy, transitions of care, and long-term care settings.

☐ Describe the importance of antimicrobial stewardship in improving public health.

SCHOLAR

☐ Educate patients and health care professionals on foundational antimicrobial stewardship concepts and empower them to practice as stewards.

13. Across the span of the entire elective curriculum, how many contact hours are devoted to antimicrobial stewardship? (e.g. a 2 hour lecture would count as 2 contact hours) ___________

14. When is antimicrobial stewardship content covered in the elective curriculum? Select all that apply.

☐ Year 1

☐ Year 2

☐ Year 3

☐ Year 4

☐ Year 5 (where applicable)

15. What pedagogies are used to teach antimicrobial stewardship content in the elective curriculum? Select all that apply.

☐ Didactic lectures

☐ Large group interactive discussions (20 or more students)

☐ Small group interactive discussions (less than 20 students)

☐ Student presentations or peer-to-peer instruction

- ☐ Simulation and role-playing
- ☐ E-learning modules (e.g. online courses and webinars)
- ☐ Project-based learning, by creation of project reports, strategic papers, and critical appraisal of literature

16. Which assessment methods are used in the elective curriculum to evaluate students' knowledge, skills and attitudes related to antimicrobial stewardship? Select all that apply.

- ☐ Multiple choice/single best answer questions
- ☐ Written assessments (e.g. essays, written answer questions, pharmacotherapy care plans)
- ☐ Oral assessments (e.g. presentations, video assignments, oral examinations)
- ☐ Assessment of group discussions
- ☐ Other, please specify: ____________

SECTION 4: Interprofessional and Experiential Education

17. Do students learn any antimicrobial stewardship content in an interprofessional setting? (e.g. with students from other faculties, such as medicine, nursing, dentistry, veterinary medicine)

- ○ Yes
- ○ No

18. Does your program offer experiential rotations with a focus on antimicrobial stewardship? (eg. >50% of student's time is devoted to antimicrobial stewardship-related activities)

- ○ Yes – Early practice experiences (EPE) are available. The experiences occur prior to the student's final year of the program.
- ○ Yes – Advanced pharmacy practice experiences (APPE) are available. These experiences occur during the student's final year of the program.
- ○ Yes – Both EPE and APPE are available.
- ○ No
- ○ Unknown

SECTION 5: Opportunities and Barriers to Enhancing Antimicrobial Stewardship Education

19. What are perceived barriers to increasing the depth and breadth of antimicrobial stewardship content in your program's curriculum? Select all that apply.

- ☐ Lack of suitable instructor(s) with content expertise
- ☐ Lack of time available in the curriculum
- ☐ Lack of a clear curriculum framework for antimicrobial stewardship (e.g. an organized plan or set of standards or learning outcomes that defines the content to be learned)
- ☐ There are other higher priority topics in the curriculum
- ☐ It is considered more relevant as a continuing education topic for practicing clinicians, as opposed to core content for undergraduate students
- ☐ It is a topic best learned in experiential rotations
- ☐ Real or perceived lack of student interest in the topic
- ☐ Other: _________________________

20. What do you perceive as facilitators to increasing the depth and breadth of antimicrobial stewardship content in your program's curriculum? Select all that apply.

☐ Having a course designated to antimicrobial stewardship
☐ More guidance on how to incorporate antimicrobial stewardship into the pre-existing curricular structure
☐ Having antimicrobial stewardship recognized as a priority by your curriculum committee
☐ Having a clear curriculum framework for antimicrobial stewardship (e.g. an organized plan or set of standards or learning outcomes that defines the content to be learned)
☐ Other: ___________________________

21. Please leave any additional comments in the box below to help us better understand your responses to the survey questions. (Optional) __________________

22. Would you be willing to share any relevant course outline(s) so that we can better understand how antimicrobial stewardship fits into your program's curriculum? (If yes, please email to jenna.sauve@uhn.ca)

○ Yes
○ No

---END OF SURVEY---
